# Testing the Feasibility and Psychometric Properties of a Mobile Diary (myWHI) in Adolescents and Young Adults With Headaches

**DOI:** 10.2196/mhealth.3879

**Published:** 2015-05-08

**Authors:** Anna Huguet, Patrick J McGrath, Michael Wheaton, Sean P Mackinnon, Sharlene Rozario, Michelle E Tougas, Jennifer N Stinson, Cathy MacLean

**Affiliations:** ^1^IWK Health CentreCentre for Research in Family HealthHalifax, NSCanada; ^2^Dalhousie UniversityDepartment of Community Health and EpidemiologyHalifax, NSCanada; ^3^Dalhousie UniversityDepartments of Community Health and Epidemiology, Pediatrics and PsychiatryHalifax, NSCanada; ^4^Nova Scotia Health AuthorityHalifax, NSCanada; ^5^Dalhousie UniversityDepartment of Psychology and NeuroscienceHalifax, NSCanada; ^6^The Hospital for Sick ChildrenChild Health Evaluative SciencesToronto, ONCanada; ^7^University of TorontoLawrence S Bloomberg Faculty of NursingToronto, ONCanada; ^8^Memorial University of NewfoundlandFaculty of MedicineHealth Sciences CentreSt John's, NLCanada

**Keywords:** headache, diary, smartphone, feasibility, psychometric properties

## Abstract

**Background:**

Headaches are prevalent among teens and young adults. Self-monitoring is essential for managing headaches and can be accomplished with the help of electronic headache diaries. An increasing number of electronic headache diaries exist, yet the absence of quality standards compromises their use for research and clinical purposes.

**Objective:**

Our goal was to develop and test the usability, feasibility, and psychometric properties of an electronic diary iPhone application for self-monitoring by adolescents and young adults with headaches.

**Methods:**

We used an iterative participatory design to develop and test our electronic headache diary. Participants aged 14-28 years old with recurrent headaches were recruited internationally. Screening and consent were conducted online. Following completion of an online pre-questionnaire, participants downloaded the diary to use in their natural environment for 14 days. An online post-questionnaire was completed following testing. The diary’s usability and feasibility were tested first and determined to be complete when improvements to the diary did not result in a statistically significant impact on indicators of feasibility and adherence. Interviews were conducted with participants of usability and feasibility testing. The psychometric properties of the diary were then tested, and a case study analysis of one participant was completed.

**Results:**

Three cycles to test the usability and feasibility were conducted. Each cycle included 11-19 unique participants ranging in age from 16 to 28 years. Following the testing period for each cycle, 15% to 25% of participants took part in the post-cycle interview. Participants perceived the final version of the diary as useful, easy to learn, and efficient to use. Psychometric properties were then tested with a sample of 65 participants (6 aged 14-17 years old; 59 aged 18-28 years old). All items in the diary had substantial between- and within-subjects variability (percent of variance for the two participant groups ranged from 20.64 to 75.60 and 23.74 to 79.21, respectively). Moreover, the Migraine Disability Assessment (MIDAS) included in the diary had adequate between-subjects reliability (R1F=0.66, RKF=0.98), but low within-subjects reliability (RC=0.51). Critical elements of the diary demonstrated adequate convergent and concurrent validity, particularly in the older age group (18-28 years). The validity of some critical elements of the diary could not be explored in the younger age group due to the small subgroup size. The case study provides an example of the potential utility of the diary.

**Conclusions:**

Our electronic headache diary was shown to be a usable and feasible self-monitoring tool when used by adolescents and young adults with headaches for 14 days. This study provides preliminary support of its psychometric properties. Our diary has the potential for helping users to better understand their headaches and, consequently, to change behaviors to improve self-management of their headaches. Its effectiveness as a component of an intervention will be the focus of future research.

## Introduction

Tension-type headache (HA) and migraine are ranked as the second and third most common diseases in the world, affecting 20.8% and 14.7% of the world population, respectively [1]. Migraine is ranked as the eighth leading cause for disability [1]. Due to the burden of these conditions, effort toward improving care is warranted.

Consistent with the International Headache Society guidelines [2], health care professionals often advise diary use to self-monitor headaches [3,4]. Self-monitoring enables recognition of temporal behavior patterns, allows individuals to become informed and actively self-manage headaches, facilitates treatment decision-making and treatment tailoring, and offers a measure of treatment efficacy [5]. Self-monitoring is particularly useful for people with recurrent headaches, whose episodes usually occur in response to unrecognized triggers [6]. Diaries can help individuals understand headache patterns and identify triggers [7], which is a basic treatment strategy for headaches [8,9]. Behavioral management of these triggers can result in fewer headaches [10]. Findings from meta-analyses indicate that behavioral interventions, usually including self-monitoring, are also effective at reducing headaches [11].

In addition to the clinical advantages of self-monitoring with headache management, diaries also have research benefits. Diaries allow researchers to test hypotheses of within-subject relations over time as an extension of prior cross-sectional research (eg, finding whether headache episodes more likely to occur in the context of a putative trigger) [12,13].

While paper diaries have long been used for self-monitoring headaches [14], advances in technology have afforded widespread use of electronic diaries (e-diaries) [10,15-18]. E-diaries offer several advantages, including increased adherence, accuracy, acceptability, and efficiency [14,18-21]. Two recent systematic reviews identified 5 e-diaries used in research [22] and 38 in Canadian mobile app stores for iOS and Android platforms [23]. The quality of these self-monitoring tools is questionable in the absence of any existing standards. Current headache e-diaries have several limitations, including not using a participatory design [24], recording insufficient data to provide understanding of headache patterns, lacking evidence that demonstrates feasibility and psychometric properties, and lacking research on the impact of these diaries on health outcomes [11,22].

Our overall goal was to create a usable, feasible, and psychometrically sound electronic headache diary for people aged between 14 and 28 years old who have recurrent headaches that also overcame some of the identified weaknesses of existing diaries. The specific objectives of this study were to: (1) test and improve the usability and feasibility of a new iPhone-based diary in terms of adherence, learnability, acceptability, efficiency, and accuracy through the use of iterative cycles; (2) test the psychometric properties (eg, reliability/convergent and concurrent validity) of this diary when used for assessment purposes; and (3) illustrate its potential utility with a case study analysis.

## Methods

### Overview

Participants were eligible if they self-identified as: (1) being aged 14-28; (2) having proficiency with English speaking, reading, and writing; (3) having a headache frequency of 2 or more episodes each month for the last 3 months; and (4) owning an iPhone with a data plan or wireless Internet access. Participants were excluded if they self-identified as having: (1) cognitive and/or developmental delays; (2) not visited a physician to exclude an organic disorder or traumatic injury as the cause for their headaches; and/or (3) significant visual impairment or blindness. Participants who consented but did not complete the pre-assessment questionnaire were also excluded.

### Concept and Development Process of the Electronic Headache Diary

**Figure 1:**
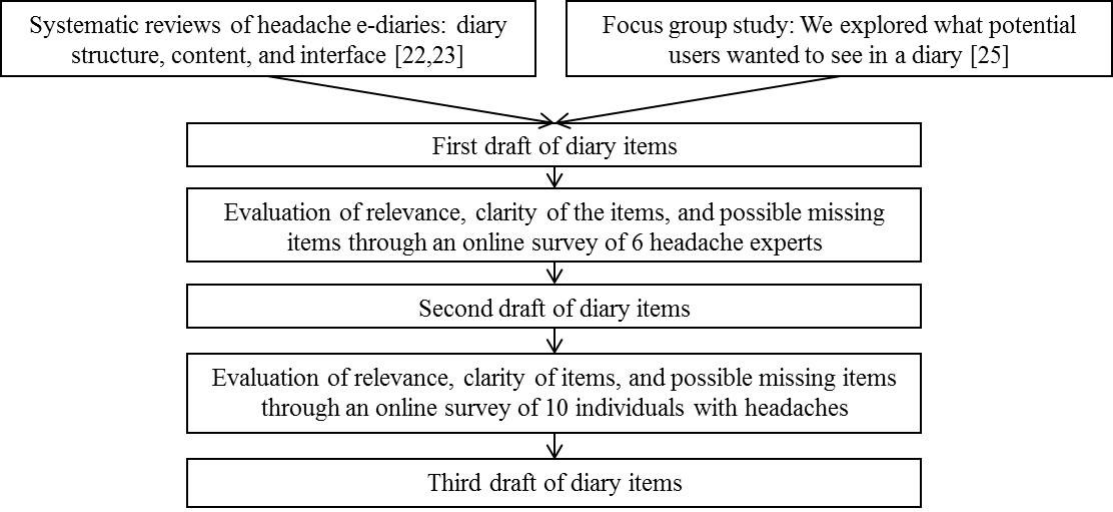
Defining the concept of myWHI diary.

Several steps were taken to create our electronic headache diary application (see Figure 1). Once diary items and features were well-defined, an iterative process that involved 3 cycles of designing, testing, reviewing, and refining the e-diary app was followed. Initially, low-fidelity paper diary prototypes, followed by high-fidelity software-based diary prototypes, were tested with volunteers in the lab. Potential end users were then involved in 3 iterative cycles of testing high-fidelity, software-based diary prototypes in participants’ natural environments. The final high-fidelity prototype was used to test the psychometric properties of the diary. The evaluation of the diary with potential end users during the iterative cycle phase is the focus of this manuscript.

### Evaluation of the Usability, Feasibility, and Psychometric Properties of the Diary

Participants involved in usability/feasibility testing and psychometric properties testing followed the same procedure unless indicated. Participants were recruited internationally through online advertisements on social networks, classified ads, and mailing lists. We used an automated Web-based system for efficient online screening and consent that closely mimicked the electronic signup process that will be followed when the application is released to the public. Potential participants were asked screening questions by the automated system. Those identified as eligible were automatically asked to provide online consent (assent and parent authorization if between 14-16 years old). If consent was provided, an online pre-questionnaire was completed. The researcher then emailed each new participant with instructions for downloading the iPhone application along with the request to use the diary for 14 days. After this testing period, an online post-questionnaire was completed and 15% to 25% of participants involved in the usability/feasibility testing were randomly selected for a 45-minute, end-of-study interview via phone or Skype. Participants involved in psychometric testing were not interviewed. Participants were reimbursed for their time. This study was approved by the IWK Health Centre’s Research Ethics Board.

## Measures

### Electronic Headache Diary

Our electronic headache diary was called the myWireless Headache Intervention diary (myWHI diary). It was designed to help users become more aware of headache symptoms and patterns. It tracks temporal, sensory, and affective aspects of headaches, and headaches’ impact on daily life, potential triggers, and coping behaviors. The diary incorporates ad-hoc and validated paper measures (ie, MIDAS/PedMIDAS and NRS-11). It includes outcome measures recommended by IMMPACT, PedIMMPACT [25-27], and guidelines for both pharmacological and behavioral clinical trials of headache [28-30] to facilitate use of the diary in a scientific trial context. The diary encourages users to complete a headache entry for every headache. First, users report occurrence by specifying start time, initial intensity, starting location, potential trigger(s), and prior symptoms (Figure 2.1). At the end of the headache, users are encouraged to report ending time, headache quality, highest pain intensity, level of unpleasantness, associated symptoms, change in headache location, and medication taken or strategies utilized to cope with the headache. At the end of each day, regardless of whether they had a headache, users are asked to enter additional information into the daily diary (eg, overall mood, hours and quality of sleep; see Figure 2.2). In addition, participants record headache impact on daily activities if a headache occurred that day. As all items are optional, users can keep track of information most relevant to them. The diary provides visual graphs of headaches over time in terms of occurrence, intensity, duration, and level of headache-related interference in daily functioning. To highlight potential causal relationships, users can also see how these parameters are related with the tracked potential triggers (Figure 2.3).

**Figure 2:**
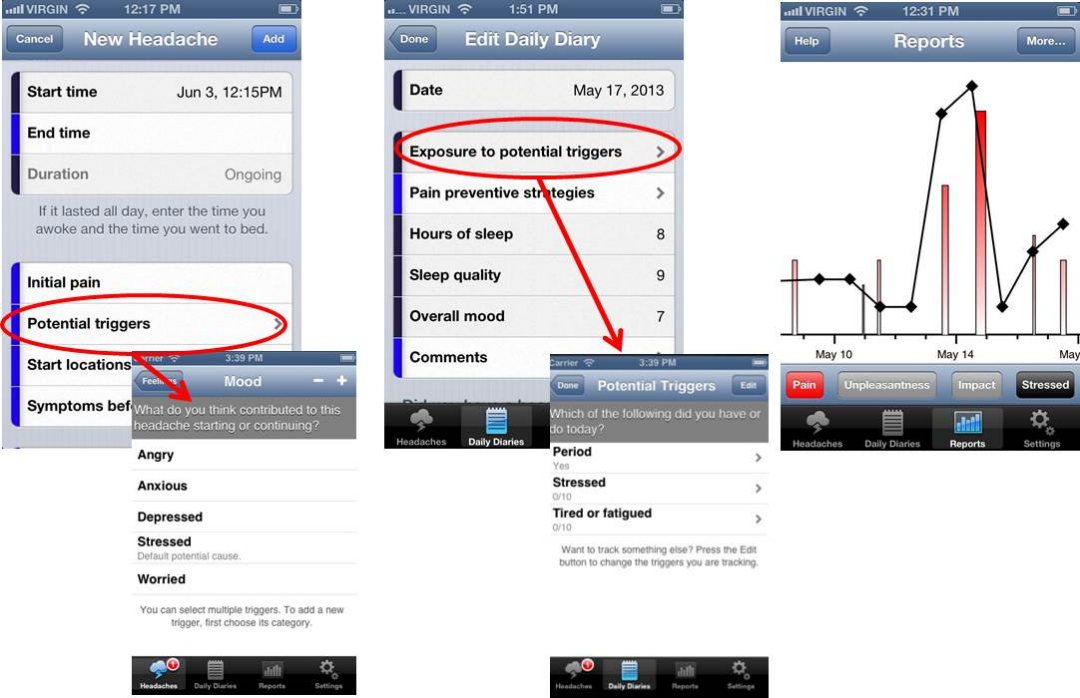
Screenshots of the myWHI diary interface. 2.1 Headache entry 2.2 Daily diary entry 2.3 Reports.

### Pre-questionnaire

A closed-ended questionnaire gathered demographics, iPhone usage, and headache characteristics. Based on headache characteristics and considering the International Headache Classification (IHC) criteria [31], participants were classified as having migraine-like headaches, tension-like headaches, mixed headaches (meeting criteria for both migraine-like and tension-like headaches), or unclassified headaches.

### Post-questionnaire

A closed-ended questionnaire was administered to participants of usability/feasibility testing and psychometric testing. The questions posed to the 2 testing groups were different, as explained below.

#### Post-Questionnaire for Usability/feasibility Testing

This questionnaire evaluated the diary in terms of helpfulness, usefulness, efficiency, visual appeal, and ability to be understood through ad-hoc questions.

#### Post-Questionnaire for Psychometric Testing

This questionnaire included the following standardized tools:

1. The Numerical Rating Scale (NRS-11): An 11-point self-report pain intensity scale commonly used. It has been psychometrically tested in children aged 8 years and above, adolescents [32], and adults [33,34].
2. The Mental Health Inventory-5 (MHI-5): A 5-item self-report measure to assess psychological distress during the past month [35]. A higher score indicates better mental health. MHI-5 has repeatedly been shown to be valid and reliable in adolescents and adults [36,37].
3. The Patient Reported Outcomes Information Measurement System (PROMIS) Short Form—Sleep Disturbance (adult version): A measure developed to assess the qualitative aspects of sleep. Its psychometric properties have been shown to be valid for adults [38]. This scale was administered to participants aged 18 years and over.
4. The PROMIS Short Form—Pain Interference (adult version): An 8-item self-report measure to assess pain-related interference with: (1) physical functioning, (2) psychological functioning, and (3) social functioning. It has been tested in adults [39]. This scale was administered to participants aged 18 years and over.
5. The Sleep/Wake Behavior Problems Scale of the Sleep Habits Survey, a scale that evaluates erratic sleep/wake behaviors [40], and the PROMIS Pediatric Short Form—Pain Interference (child version), an 8-item self-report measure to assess pain-related interference with functioning [41], were administered to participants between age 14 and 17 years old. However, this information was not used in our analyses due to the reduced number of participants between the ages of 14 and 17 years old (see results).

### End-of-study interview

A semi-structured interview administered to 15% to 25% of participants involved in each cycle of usability/feasibility testing was facilitated by one researcher guided by a script. The questions focused on the participants’ experiences using the diary, including barriers, usefulness, burdens, goals, and suggestions for improvement.

### Data Analytic Strategy

#### Usability and Feasibility Testing of the Diary

Analysis was exploratory in nature. Using SPSS 20 predictive analytics software, descriptive statistics (median and range) were calculated for pre- and post-assessment data. Usage of the diary was automatically collected from the system. Mann-Whitney U and Chi-square tests were used to evaluate differences between consecutive testing cycles for continuous and categorical variables used as indicators of feasibility and adherence. We used non-parametric methods and report the median (mdn) instead of the mean because of our small sample size. Qualitative data collected through end-of-study interviews were transcribed, coded, and analyzed using an inductive thematic analysis [42].

#### Psychometric Properties Testing of the Diary

We hypothesized that the diary would be reliable and the most essential components of the diary would be valid. We hypothesized that convergent construct validity would be supported by high correlations between the data derived from the items of the diary that assess headache occurrence, intensity, unpleasantness, mood, and sleep and headache impact, with data obtained through questions asking for the same information retrospectively mostly using well-established single-point measures. We also hypothesized that if the most essential parts of diary had concurrent validity then a good number of variables collected through the diary would be correlated with other single-point measures that assess related constructs. Specifically, we hypothesized that the total number of headache episodes, total headache time, highest headache intensity, and unpleasantness of headaches would be moderately to strongly related to levels of pain-related interference, sleep-related impairment, and emotional functioning as assessed retrospectively through well-established single-point measures.

Reliability of most variables measured by the diary was assessed using generalizability theory analysis. Following Cranford et al [43] we used generalizability theory to decompose the variance of daily variables and to calculate reliability estimates using VARCOMP procedures in SPSS 20. For single-item variables, variance was decomposed into person, day, and person-by-day components. The person component represents the between-subjects portion of the variance that remains stable across all 14 days, and the person-by-day component represents the within-subjects component that varies by day. Thus, a variable with a high proportion of person variance is highly stable over time, which is analogous to having high test-retest reliability. Because MIDAS/PedMIDAS, which measures the impact of headache on the subject’s daily functioning, is a multi-item measure, the variance was decomposed into person, day, item, person-by-day, person-by-item, day-by-item, and error variability. This information is used to calculate 3 estimates of between- and within-subjects reliability [43]. R_1F_ is equivalent to calculating Cronbach’s alpha for each of the 14 days separately, and taking the average of all 14 alphas. R_KF_ values are equivalent to averaging all 14 days of data into a single composite index, then calculating Cronbach’s alpha once. R_C_ values represent within-subjects reliability, which represents the precision of the measure for measuring systematic variance from day-to-day.

The convergent construct validity and criterion validity of the diary were assessed by correlating several items of the diary with well-established 1-point measures. Because diagnostics (ie, kurtosis, skewness, histograms, P-P plots) suggested many variables were non-normally distributed, we used Spearman’s rank-order correlation coefficient (r_s_). Cohen’s criterion (small = 0.10; medium = 0.30; large = 0.50; Cohen et al [44]) was used to interpret effect sizes. Variables from the diary were combined across all 14 days into a single value per participant using various functions (eg, sums, averages, maximum value) to facilitate comparison with variables measured only once. A full list of all convergent and criterion validity tests can be found in Table 7. The minimum required sample size to achieve 80% power assuming to find at least medium-to-large effect size (r_s_=0.40) and an alpha of 0.05 was 46 people.

### Case Study Analysis

A representative example of a participant of psychometric properties testing was selected.

## Results

Table 1 provides the descriptive statistics of the participants involved in evaluation of the diary. In the usability/feasibility testing cycles, the majority of participants were young adult females with median ages of 24 to 25 years old (range, 16-28 years). The most frequent type of headache was migraine-like headache. In psychometric testing, the median age was similar, 22 years old (range, 14-28); only 6 of the 65 participants were 14 to 17 years old, and 59 were 18 to 28 years old. As in usability/feasibility testing, the majority of participants were female and the most common type of headache was migraine-like headache.

**Table 1:** Descriptive statistics of participants involved in the evaluation of the myWHI diary.

		Usability/Feasibility testing (n=43)			Psychometric testing (n=65)
		Cycle 1 (n=11)	Cycle 2 (n=19)	Cycle 3 (n=13)	
Age					
	Median years (range)	25 (16-28)	24 (16-28)	25 (20-28)	22 (14-28)
Gender					
	Males	1	4	1	9
	Females	10	15	12	56
Headache diagnosis^a^					
	Migraine-like headache	9	14	11	46
	Tension-like headache	0	0	0	3
	Mixed headache	0	0	0	0
	Unclassified headache	1	1	0	1
	>1 headache diagnosis	1	4	2	8

^a^ *Migraine-like headache*: Headache with at least 2 of the following: (a) unilateral location, (b) pulsating quality, (c) moderate or severe pain intensity, (d) aggravation by or causing avoidance of routine physical activity. And during the headache, experiencing one of the following: (a) nausea and/or vomiting, (b) photophobia and phonophobia. *Tension-like headache*: Headache with at least 2 of the following: (a) bilateral location, (b) pressing or tightening (non-pulsating) quality, (c) mild or moderate intensity, (d) not aggravated by routine physical activity such as walking or climbing stairs. And during the headache experiencing both: (a) no nausea or vomiting, (b) no more than one of photophobia or phonophobia. *Mixed headache*: Headache meeting all criteria to be classified as both migraine-like and tension-like headache. *Unclassified headache*: Headache that does not meet all clinical categories outlined in the IHC criteria and defined above to classify headaches.

**Figure 3:**
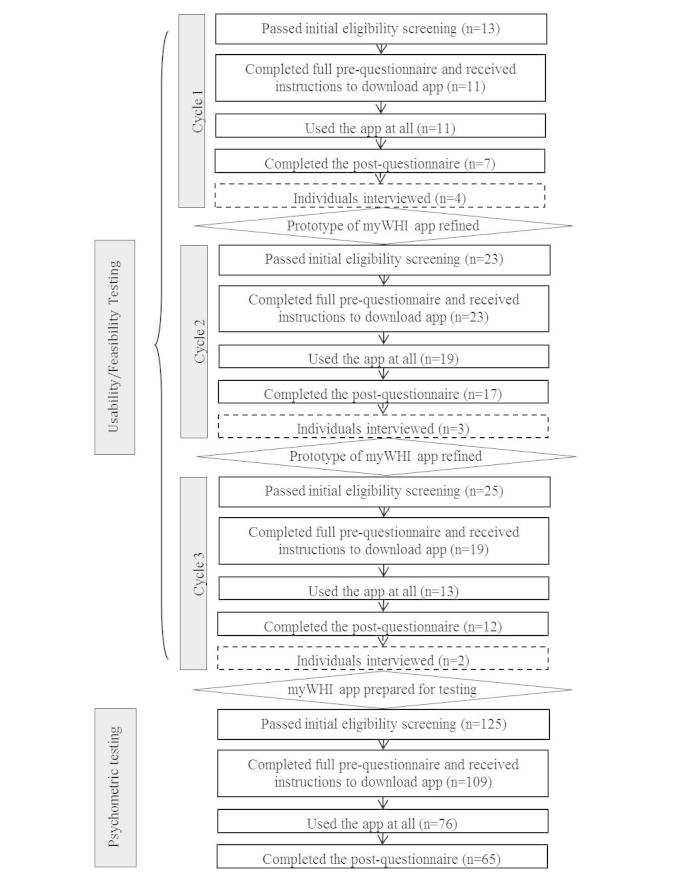
Flowchart of participation in the evaluation of the myWHI diary.

Figure 3 shows the flowchart of participation in the evaluation of the diary. Results of usability/feasibility testing are based on participants who used the diary application at least once during Cycle 1 (n=11), Cycle 2 (n=19), and Cycle 3 (n=13). Results of psychometric testing are based on participants who completed the post-questionnaire (n=65).

### Usability and Feasibility Testing of the Diary

#### Adherence

Table 2 shows indicators of adherence. In Cycle 1, headache entries tended to be entered a long time after reported start time (mdn=13.59 h; range, 1.73-101.28 hours). Headaches were often not recorded on the actual day that the episode was reported to have occurred. However, most important headache items were answered with each entry. A minority of participants completed all 14 daily diary entries (18%, n=2) or 75% of the 14 daily diary entries (18%, n=2). The items encouraged to be answered every day were usually entered the day that the daily entry was made. Following Cycle 1, changes were made to the diary to make the application clearer, which we expected would increase adherence. The most significant changes are shown in Table 4.

In Cycle 2, statistically significant improvements in adherence with headache entries were found following refinements to the first prototype. Participants in Cycle 2 completed their headache entries closer to the time pain began than did participants in Cycle 1 (Cycle 2 mdn=3.83 h; range, 0.09-19.92 hours, vs Cycle 1 mdn=13.59 h; U=32.00, z=-3.01, *P*=.003). Adherence with the daily diary entries also improved, but did not reach statistical significance (26% of participants, n=5, completed all 14 daily entries in Cycle 2 vs 18% of participants, n=2, in Cycle 1; χ^2^_(1)_=0.26_,_ *P*=.69; 53% of participants, n=10, completed 75% of the 14 daily diary entries in Cycle 2 vs 18% of participants, n=2, in Cycle 1; χ^2^_(1)_=3.44_,_ *P*=.12). As observed in Cycle 1, participants in Cycle 2 also tended to answer all of the items when completing a headache or daily entry (see Table 2). Following Cycle 2, minor changes were made to the diary primarily to increase adherence (see Table 4).

In Cycle 3, the level of participant adherence utilizing the diary for headache entry remained acceptable with no statistically significant differences found between Cycle 2 and Cycle 3 (see Table 2). As observed in Cycle 2, the majority of participants’ headache entries during Cycle 3 were made on the same day that the episode occurred. Once participants created the headache entry, they tended to report initial information about their headache right away. The level of adherence of participants utilizing the diary for entering the daily diary entries was not statistically different from Cycle 2. Participants completed the majority of daily entries in real-time with only a minority of daily entries entered retrospectively. Because significant improvements in feasibility indicators of the diary were not observed in Cycle 3, we decided not to make further changes. This was the final version of the diary used to test the psychometric properties.

**Table 2:** *.* Median level of adherence with the myWHI diary for Cycles 1-3^a^.

			Cycle 1 (n=11)	Cycle 2 (n=19)	Cycle 3 (n=13)
Headache (HA) entries					
	HA entries made the same day that HA was reported to happen, per participant		57% (0%-100%)	100%^c^ (23%-100%)^b^	82%^c^ (31%-100%)^c^
	Times that the following items were answered when entering an HA episode, per participant				
		Initial intensity	100% (56%-100%)	100% (0%-100%)^c^	100% (50%-100%)^c^
		Start location	100% (56%-100%)	100% (25%-100%)^c^	100% (0%-100%)^c^
		Potential triggers	99% (50%-100%)	100% (0%-100%)^c^	85% (0%-100%)^c^
		Highest intensity	100% (56%-100%)	100% (50%-100%)^c^	100% (50%-100%)^c^
Daily diary entries					
	Daily diary entries made		42.8% (0%-100%)	78.57% (21.43%-100%)^c^	85.71% (0%-100%)^c^
	Daily entries made retrospectively for another day, per participant		22% (0%-50%)	0% (0%-50%)^c^	14% (0%-73%)^c^
	Times that the following items were answered when entering a daily diary, per participant				
		Hours of sleep	100% (20%-100%)	100% (86%-100%)^c^	100% (29%-100%)^c^
		Sleep quality	100% (80%-100%)	100% (79%-100%)^c^	100% (29%-100%)^c^
		Overall mood	100% (100%-100%)	100% (77%-100%)^c^	100% (29%-100%)^c^
		Pain interference	61% (0%-100%)	75% (0%-100%)^c^	100% (50%-100%)^c^
		Pain unpleasantness	100% (56%-100%)	100% (50%-100%)^c^	100% (40%-100%)^c^
		Pain qualities	100% (56%-100%)	100% (50%-100%)^c^	100% (50%-100%)^c^

^a^Reported values are the median percentage values across Cycles. The ranges of all reported values are presented in parentheses.

^b^Difference between values for consecutive Cycles (Cycle 1 vs Cycle 2; Cycle 2 vs Cycle 3) is statistically significant (*P*<.05).

^c^Difference between values for consecutive Cycles (Cycle 1 vs Cycle 2; Cycle 2 vs Cycle 3) is not statistically significant.

### Usage of Diary Features

Table 3 summarizes how participants used the features of the diary.

**Table 3:** Usage of diary features for Cycles 1-3.

		Cycle 1 (n=11)	Cycle 2 (n=19)	Cycle 3 (n=13)
Web reports				
	% (#) visited reports	N/A	0% (0)	0% (0)
Reminders				
	% (#) disabled reminders	9% (1)	5% (1)	7% (1)
	% (#) modified reminder time	36% (4)	47% (9)	31% (4)
	Range of reminder time	5:00pm-10:30pm	9:00pm-11:00pm	7:00pm-10:00pm
Customizable list of potential triggers to record daily				
	% (#) edited default trigger list	73% (8)	58% (11)	54% (7)
	Mdn # (range) of potential triggers concurrently recorded, per participant	2 (0-4.2)	3.62 (2-21.5)	3.67 (2-9.2)
	Top 5 most common selected triggers per Cycle: % (#) of participants who tracked each trigger	Stressed: 91% (10)	Stressed: 100% (19)	Stressed: 85% (11) ^a^
		Tired/fatigued: 83% (9)	Tired/fatigued: 100% (19)	Tired/fatigued: 77% (10)
		Period: 37% (4)	Period: 74% (14)	Period: 77% (10)
		Lack of sleep: 27% (3)	Computer use: 21% (4)	
		Caffeine, less than usual: 27% (3)	Missing meal/ hunger: 21% (4)	
Comments section				
	% (#) of participants who used the headache comments	45% (5)	50% (9)	46% (6)
	Mdn % (range) times the headache comments section used	22.22% (14.29%-50%)	40% (16.67-100)	50% (9.09-60)
	% (#) of participants who used daily diary comments	45% (5)	58% (11)	64% (7)
	Mdn % (range) times daily diary comments section used	33.33% (14.29%-100%)	27.27% (7.69%-80%)	14.28% (7.14%-50%)

^a^For Cycle 3, there were only 3 top triggers.

### Learnability, Acceptability, and Efficiency

#### Information Collected Through the Online Post-Questionnaires

**Figure 4:**
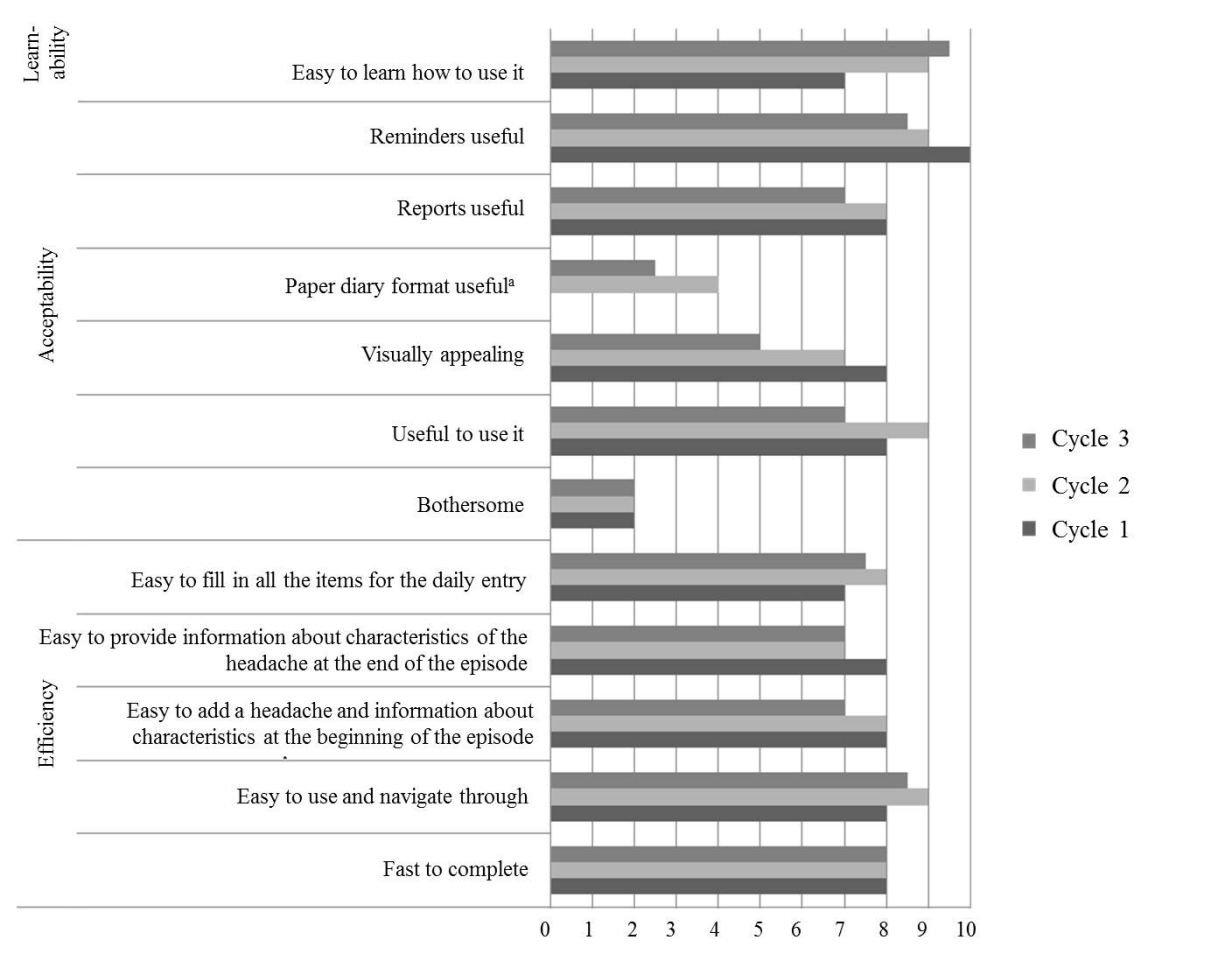
Participants’ feedback on attributes of the myWHI diary (mdn values). aNo participant used the paper-format diary in Cycle 1.

Figure 4 shows participants’ opinions on attributes of the diary. In Cycle 1, 7 of 11 participants completed the post-questionnaire. In terms of acceptability, all participants reported that they would recommend this diary to others. Five participants (71%) expressed interest in continuing to use the diary (eg, for 6 more weeks). Six participants (55%) continued using the diary for 2 weeks following their 14-day trial, with 3 (27%) continuing to use it beyond that. This behavior occurred in the absence of any incentives or encouragement. In terms of efficiency, the headache diary item that was repeatedly reported to be the most difficult to complete was “potential triggers” (n=4, 57%).

In Cycle 2, 17 of 19 participants completed the post-questionnaire. Participants’ opinions on the feasibility of the diary remained positive and improved when contrasted with opinions in Cycle 1 (Figure 4). The levels of acceptability and efficiency between the first 2 cycles were not statistically different, with the exception of fewer participants reporting trouble recording “potential triggers” for headache entries during Cycle 2, the item with the most problems for participants in Cycle 1 (Cycle 1: 57%, 4 of 7 participants vs Cycle 2: 0%, 0 of 19 participants; χ^2^_(1)_=11.66, *P*<.001). For learnability, participants in Cycle 2 reported the diary to be easier to learn how to use than did participants in Cycle 1 (z=-2.22, *P*=.03).

In Cycle 3, 12 of 13 participants completed the post-questionnaire. The learnability, acceptability, and efficiency of the diary remained as positive as users’ experience during Cycle 2, and no statistically significant improvements were found (Figure 4).

#### Information Collected Through the End-of-Study Interviews

In Cycle 1, 4 participants were interviewed. Thematic analysis revealed 3 distinct themes:

1. Poor understanding: Many participants neglected to use features of the diary because they either did not know how the features worked, or were unaware that they existed (eg, participants were unaware that they could customize the triggers tracked on a daily basis). This lack of understanding was the prevailing message of the first cycle. As a potential solution, participants suggested the addition of tutorials.

2. General endorsements, likes, and dislikes: Participants felt satisfied with the diary and perceived it as useful because it taught them what to pay attention to; helped increase awareness of headaches, identification of triggers, and effectiveness of medications; helped guide self-care behaviors; and helped in reporting headaches to physicians and family. Participants felt that the diary was easy to use overall, but identified some difficult and confusing situations, such as: setting the start and end time of headaches as wake and sleep times, respectively; including not applicable items (eg, symptoms before and after the headache) for those with constant headache; having to develop the habit of using the diary; identifying potential headache triggers within the diary’s hierarchical presentation; and the slowing of the application due to network connections.

3. Suggestions for improvement: To improve ease of use, participants suggested: setting default answers to daily items, allowing participants to remove unused items, adding more reminders to complete either ongoing headache entries or daily entries, and providing the most frequently entered options at the top of lists to expedite data entry. They also suggested providing additional reports and adding the ability to export diary data.

In Cycle 2, 3 participants were interviewed with 3 distinct themes identified:

1. Good understanding: Participants demonstrated a good understanding of the functionality of the application. They found the tutorials that were added after Cycle 1 to be clear and useful.

2. General endorsements, likes, and dislikes: Participants found the application easy to use, relevant, and useful. They reported that using the application gave them a more accurate idea of their headaches (ie, type of pain, intensity, and potential triggers). However, they did not speak highly of some sections or functionalities, such as: no interviewed participant reported using the Web-based reports that were added after Cycle 1, instead emphasizing the convenience of reviewing reports on the phone and indicating that they were likely to consult the reports more often after using the diary for a longer period of time. They did consult the iPhone reports, but not frequently. Participants also disliked being unable to enter daily diary entries retrospectively, being unable to enter daily entries that extended past midnight without starting a new day, or having to enter day-long headaches for constant headache.

3. Suggestions for improvement: For ease-of-use improvements, participants suggested: adding the ability to be reminded about incomplete items in headache entries and the ability to see the day of the week when entering dates.

In Cycle 3, 2 participants were interviewed and the following theme was identified:

1. General endorsements, likes, and dislikes: Both participants liked the application, reporting that it was clear, easy to use, and useful. Despite their satisfaction with the overall application, a few features were not always perceived as useful. Whereas 1 participant used the comment section throughout headache episodes to track perceived changes, the other reported that the section was not useful. While 1 participant found the reports appealing and easy to interpret, the other found them confusing. Other aspects that were raised as a source of dissatisfaction or not used were: the loading time, the difficulty deciding what daily triggers to track due to the large number of available options, and the Web-based reports. They expressed again that access to the reports from within the mobile application would be more beneficial than accessing them through the Web.

### Summary of Diary Changes

As part of the iterative design process, the diary was refined following Cycles 1 and 2 based on participant feedback. Table 4 summarized the most important changes made.

**Table 4:** Most important changes made to the diary during testing.

Reason for change	Change made
Cycle 1	
To improve learnability	Added automatic help/instruction slides to explain how the application works when the user first launches the application. Added help buttons to the headaches, daily diary, and reports tabs to view the help/instructions slides again at any time. Moved the feature that allows users to customize the list of potential triggers from the settings area to the daily diary entries to make it clearer that the list can be modified at any time. Added detail text (ie, “This is a default”) to the triggers setup as a default for tracking daily exposure, so that users can easily recognize the suggested default triggers. Added a “More” button to the reports tab to explain accessing the Web-based reports. MyWHI 2.0 is the first version of the application with Web-based reports.
To improve efficiency	Added a “Frequently used” category containing the 5 most-reported triggers. Updated the order of medications to include the most recently used at the top, followed by those never taken ordered alphabetically. Modified several lists of answer options to be sorted alphabetically.
To improve accuracy	Modified the date selection to disallow selection of future dates and times.
To improve acceptability	Added more PC-accessible-only reports to provide the user with more information from their entered data. Added question text for “Potential triggers” to the top of the screen when searching for the appropriate response option within the hierarchical tree structure to help users to keep the question of what might trigger a headache in mind.
Cycle 2	
To improve adherence	Added a badge to the headaches tab to indicate ongoing headaches. Added an alert message to remind participants to create subsequent daily diary or headache entries sooner, if a diary entry is created for a previous day or a headache is entered more than 3 hours after the reported start time. Added an alert to the “Duration” item, which is automatically reported by the system once the user has entered “Start time” and “End time.” The item turns red if the headache duration is a negative value. Added a reminder message that shows up if headache duration exceeds 24 hours. The message reminds the participant that headaches lasting more than 1 day should be recorded as a separate headache for each day.
To improve learnability	Added a reminder for the user to look at the reports. This shows up after the 5th, 10th, 15th, etc. daily diary entry is added until the user views reports.
To improve acceptability	Removed the restriction on creating a diary entry if one already exists. This was preventing users from adding diary entries for previous days. Revised the date format in headaches and daily diary entries to “Monday, June 6” instead of “June 6.”
To improve efficiency	Changed medication search to prioritize medications that contain the search string at the beginning (eg, searching “ty” brings up “Tylenol” rather than “Atyopanex”).
To improve accuracy	Fixed bugs (eg, fixed a bug that caused an error when saving empty MIDAS responses).

### Psychometric Properties Testing of the Diary

The 65 participants completed a mean of 10.32 daily diary entries (SD=4.94) during the 14-day period. A total of 33 participants (65%) completed all 14 daily diary entries and 44 (67%) completed more than 75% of the daily diary entries.

Generalizability theory results for single-item measures recorded using the NRS-11 scale are located in Table 5. The largest portion of variance for headache start intensity, maximum intensity, and duration was explained by person variability (62.79% to 75.60%) suggesting that these variables are highly stable across individuals across 14 days. In contrast, unpleasantness, amount of sleep, quality of sleep, and mood was primarily characterized by person-by-day variability (60.27% to 79.21%) suggesting that these variables tended to vary substantially from day to day. However, all variables had substantial person and person-by-day variability suggesting that these variables capture both trait-like individual differences and state-like daily fluctuations. Reliability for PedMIDAS was not calculated due to the small sample size of users between the ages of 14 and 17 years old. Generalizability theory results for MIDAS (Table 6) shows that MIDAS has substantial person and person-by-day variance, but also has a substantial amount of measurement error. The various estimates of reliability were excellent (R_KF_=0.98), adequate (R_1F_=0.66), and somewhat low (R_C_=0.51), depending on the measure used. These results suggest that MIDAS has good reliability if data from all 14 days are aggregated to create a single estimate for a person, but may not be suitable for measuring day-to-day variations in headache impact.

**Table 5:** Variance components for single-item measures of the diary.

Source of variance		Person^a^	Day^b^	Person-by-day^c^	Total^d^
Start intensity					
	0-10 NRS	2.495	0.077	1.234	3.806
	% overall variance	65.55%	2.03%	32.42%	100.00%
Highest intensity					
	0-10 NRS	3.076	0.128	1.694	4.899
	% overall variance	62.79%	2.62%	34.59%	100.00%
Duration					
	Difference between starting and ending time (in minutes)	102,960.884	891.587	32,333.475	136,185.946
	% overall variance	75.60%	0.65%	23.74%	100.00%
Unpleasantness					
	0-10 NRS	2.387	0.005	3.627	6.019
	% overall variance	39.654%	0.075%	60.27%	100.00%
Amount of sleep					
	Hours of sleep	0.969	0.007	3.332	4.207
	% overall variance	20.64%	0.16%	79.21%	100%
Quality of sleep					
	0-10 NRS	1.276	0.000	3.486	4.762
	% overall variance	26.80%	0%	73.20%	100%
Mood					
	0-10 NRS	0.955	0.002	3.023	3.980
	% overall variance	23.99%	0.06%	75.95	100%

^a^Person = variance due to between-person differences across all days.

^b^Day = variance due to differences between days across all persons.

^c^Person-by-day = variance due to between-person differences at different days.

^d^Total = sum of all variances.

**Table 6:** Variance components for multi-item measures of the diary.

Source of variance	Headache impactMIDAS^a^	% overall variance
Person^b^	0.060	27.91%
Day^c^	0.002	0.93%
Item^d^	0.017	7.91%
Person-by-day^e^	0.034	15.81%
Person-by-item^f^	0.005	2.33%
Day-by-item^g^	0.000	0.00%
Error^h^	0.097	45.12%
Total	0.22	100.00%
Between Subjects Reliability (R_1F_)^i^	0.66	
Between Subjects Reliability (R_KF_)^i^	0.98	
Within Subjects Reliability (R_C_)^i^	0.51	

^a^PedMIDAS measure was not explored due to the small sample size that completed this measure.

^b^Person = variance due to between-person differences across all days and items.

^c^Day = variance due to differences between days across all persons and items.

^d^Item = variance due to responses to scale items across all persons and days.

^e^Person-by-day = variance due to between-person differences at different days across all items.

^f^Person-by-item = variance due to between-persons differences in responses to scale items across all days.

^g^Day-by-item = variance due to differences between days in responses to scale items across all persons.

^h^Error = Person x Day x Item interaction plus random error (unknown sources of variance).

^i^R_1F,_ R_KF,_ and R_C_ are forms of internal consistency calculated using formulas from Cranford et al (2006) [43].

Table 7 summarizes the correlations used to validate several sections of the diary. Although we administered The Sleep/Wake Behavior Problems Scale of the Sleep Habits Survey [40] and the child version of the PROMIS Pediatric Short Form—Pain Interference [41] this information was not used in our analyses due to the reduced number of participants between the ages of 14 and 17 years old (n=6). All convergent construct validity correlations were statistically significant, and 4 of 5 had large effect sizes (r_s_>|0.50|). These correlations ranged from -0.26 (average sleep quality recorded with the PROMIS Sleep Disturbance scale) to 0.81 (highest headache pain recorded on the diary with retrospective recall of highest intensity). A total of 8 of the 12 concurrent validity correlations were statistically significant with medium to large effect sizes, ranging from -0.22 (average headache unpleasantness and MHI-5 emotional functioning) to 0.55 (highest headache pain intensity on the diary and the adult PROMIS Pain Interference scale). Due to a very small sample of adolescent participants (n=6), 10 planned correlations using adolescent measures (for participants between 14 and 17 years old) were omitted.

**Table 7:** Convergent and concurrent validity Spearman rank-order correlations for sections of the diary.

myWHI diary variables	Convergent construct validation		Concurrent validation				
*How it was calculated*	Post- questionnaire measures	r_s_^a^	myWHI diary criteria			Post- questionnaire measures	r_s_^a^
Headache entries							
Occurrence							
*Total number of recorded HA episodes*	Recall of number of HA episodes in past 14 days	0.630^e^ (n=63)	Pain interference^a^			PROMIS Pain Interference	0.293^c^ (n=59)
			Emotional functioning			MHI-5	-0.196 (n=57)
			Sleep-related impairment^b^			PROMIS Sleep Disturbance	0.165 (n=59)
Duration							
*Sum of all headache durations in minutes*	--	--	Pain interference^b^			PROMIS Pain Interference	0.422^d^ (n=51)
			Emotional functioning			MHI-5	-0.360^d^ (n=56)
			Sleep-related impairment^b^			PROMIS Sleep Disturbance	0.192 (n=51)
Highest pain							
*Recorded worst pain intensity ratings*	Recall of worst pain intensity on a NRS-11	0.809^e^ (n=57)	Pain interference^b^			PROMIS Pain Interference	0.549^d^ (n=52)
			Emotional functioning			MHI-5	-0.341^d^ (n=52)
			Sleep-related impairment^b^			PROMIS Sleep Disturbance	0.369^d^ (n=52)
Unpleasantness							
*Average of the unpleasantness ratings*	--	--	Pain interference^b^			PROMIS Pain Interference	0.519^d^ (n=50)
			Emotional functioning			MHI-5	-0.221 (n=55)
			Sleep-related impairment^b^			PROMIS Sleep Disturbance	0.430^d^ (n=50)
Daily diary entries							
Sleep quality^b^							
*Average of sleep quality ratings*	PROMIS Sleep Disturbance	-0.264^c^ (n=56)		--	--		--
				--	--		
Overall mood							
*Average of overall mood ratings*	MHI-5	0.652^e^ (n=62)		--	--		--
Headache impact^b^							
*Average of MIDAS scores*	PROMIS Pain Interference	0.693^e^ (n=45)		--	--		--
				--	--		--

^a^Coefficients represent Spearman rank-order correlations (r_s_).

^b^Only participants aged between 18 and 28 years old were considered for these analyses.

^c^ *P*<.05

^d^ *P*<.01

^e^ *P*<.001

### Case Study Analysis

**Figure 5:**
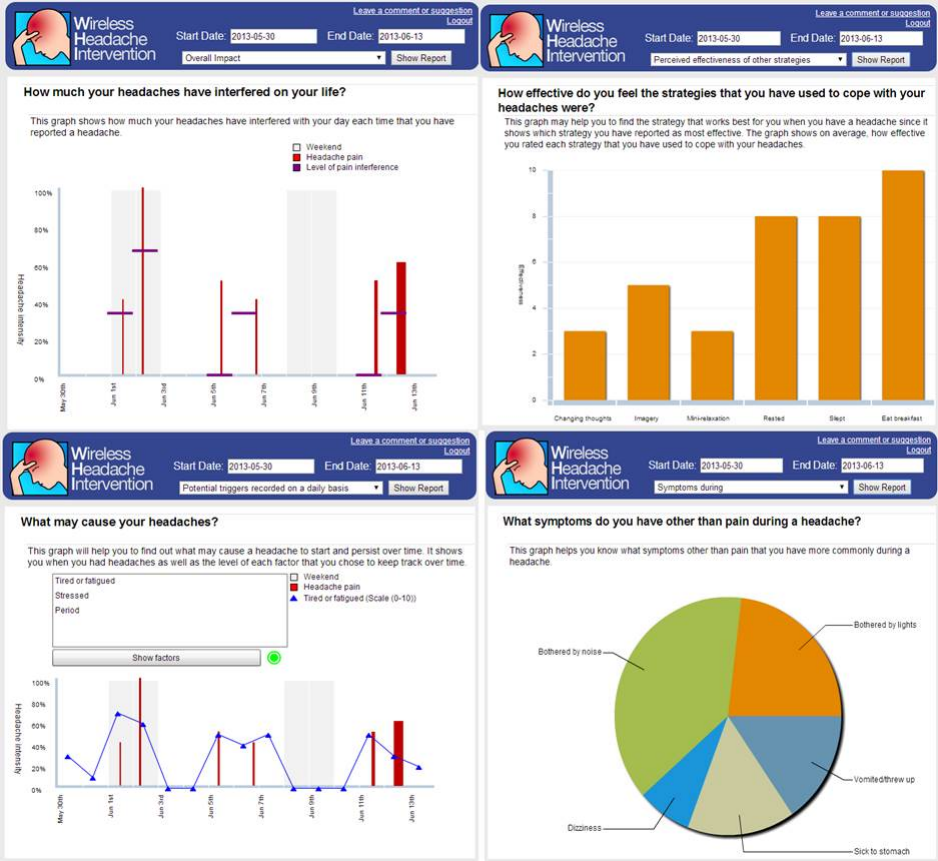
Screenshots of the Web-based reports of one of the participants.

A case study analysis of 1 participant of psychometric properties testing was conducted. The results are presented to illustrate how it is possible to improve the quality of self-reported data, consequently helping users and health care professionals to better understand headaches and improve health care decisions. Figure 5 shows screenshots of the Web-based reports of a 21-year-old female participant. This participant experienced migraine-like headaches, and recorded 6 episodes mainly of moderate headache pain over the 14 days. Most episodes lasted less than 1 hour, 2 episodes lasted around 2 and 8 hours, respectively, and caused mild to moderate interference in her life. The participant reported symptoms such as dizziness or hearing changes before the onset of half of the episodes, and often reported accompanying symptoms such as vomiting and light or noise sensitivity. The participant daily kept track of potential triggers, including: menstrual period, stress, and tiredness. There may be relationships between her stress and the occurrence of headache episodes, but it is too premature to extract conclusions from these limited observations. Besides taking ibuprofen on 2 occasions, which did not seem to help ease the pain in a timely manner, the participant reported different strategies to cope with her headache episodes, and perceived eating breakfast, rest, and sleep as the most effective. However, because of the limited duration of using the headache diary, again no conclusions about the effectiveness of these strategies can be drawn, however, this may be clinically helpful.

## Discussion

### Principal Results

After 3 iterations of designing, testing, reviewing, and refining, we created a feasible electronic headache diary. At the end of this iterative process, young adults with headaches perceived the application as useful, easy to learn, and efficient to use. Although adherence with the final version evaluated in Cycle 3 was not perfect, most participants made active use of the application throughout the testing period. The option for participants to omit items when completing the diary could have had drawbacks to this research by threatening maintenance of internal consistency while diminishing the usefulness of the diary in clinical settings. However, participants typically provided an answer to each item included in the diary when either completing a daily entry or recording a headache episode during the testing period. Moreover, most participants completed a majority of the daily entries and recorded most headache episodes on the day they occurred. Substantial differences have been observed in levels of adherence with electronic headache diaries, with rates of 75%-98% [22]. There is no consensus on the acceptable minimum adherence level in self-monitoring systems. The ultimate goal when developing a diary such as this is to provide individuals with headaches (and their health care providers) with the greatest insight into their condition while imposing minimum time and effort on maintaining a diary. This delicate balance poses a challenge. It is possible that the comprehensiveness of our application, although recognized as a strength by most participants, may have reduced adherence due to the time and effort required to complete all of the items. However, it may also take time to develop a habit of using the diary. We found that 35% of participants were still creating diary entries 2 weeks after the conclusion of their 14-day participation, and 18% created more diary entries after the study period than they had within it. This suggests that level of adherence with the diary may be maintained or even improved over time. This is most apparent with the Web-based reports, which can provide enhanced value after regular use of the application over time. It is challenging to motivate users to regularly enter data when the benefits are uncertain and meaningful reports may be weeks or even months away. Therefore, despite the possible issues with the time and effort required due to the comprehensiveness of our application, we do not plan to simplify this application, mainly because simplicity does not automatically imply that this will help patients improve adherence, and most importantly, we do not know the minimum level of adherence required to achieve better outcomes. In addition to the observed levels of participant adherence when filling in the diary, we also observed that the features incorporated in the diary (ie, the reminders, the comment sections, and the customizable list of potential triggers for the users to track their exposure on a daily basis and explore their potential relevance) were commonly used by the participants, with the exception of the Web-based report generation system that was external to the application.

We assessed reliability of measures included in the final version of the diary using generalizability theory analyses. For single-item measures, these analyses revealed that headache intensity and duration measures were primarily trait-like variables that were highly stable over time. In contrast, headache unpleasantness, amount/quality of sleep, and mood measures were more state-like, and varied considerably from day-to-day. The MIDAS had excellent internal consistency when all 14 days are combined together into a single composite variable; however, it does not appear to be a good measure for reliably measuring variability in headache-related interference from day to day. This said, poor within-subjects reliability is typical of many measures that otherwise have excellent psychometric properties [43]. Consequently, we recommend that patients and health care providers who use the diary not to examine day-to-day fluctuations in MIDAS scores. Instead, they should calculate an overall index of headache-related interference by averaging MIDAS scores across many measurement occasions and look for changes over longer periods of time using small-N, A-B-A designs. Finally, we also explored convergent and criterion validity for some critical elements of the myWHI diary, especially when used by the oldest subgroup.

### Strengths and Weaknesses of the Mywhi Application

The myWHI diary overcomes several drawbacks identified in the electronic diaries described and used in the scientific literature [22] or available on the market [23]. It allows users to track information for both headache and headache-free days, and has undergone formal usability, feasibility, and psychometric testing. Unlike the myWHI diary, the usability, feasibility, and reliability of current headache diaries are often unknown. Available diaries have been fundamentally designed to only log headaches and the main variables associated with them (ie, intensity, duration, and timing). Diaries often do not collect information in the absence of headaches, as they are event-contingent, making it difficult for the user (or health care professionals) to understand what is precipitating the headaches, and what strategies may prevent new headaches; the digital headache diary (DHD) is one exception [45]. The myWHI diary meets the criteria, which our team defined a priori, for an “ideal” diary application; it is intended to help individuals with headache to better understand and manage their headaches, while providing relevant data to health professionals [23]. The myWHI diary was created with the help of headache experts, it measures clinically relevant headache variables, it allows customization to make the application clinically relevant to the individual user, and it generates reports displaying relationships between variables.

Despite the high levels of satisfaction with the application, we also identified weaknesses. There were weaknesses with our Web-based report generation system that is external to the application, which was not used by the participants. Incorporating a robust set of reports directly in the mobile application is something to consider. The relatively slow responsiveness of the application influences user experience; we stored data on a remote server for research purposes, which introduced delays users are not accustomed to when using mobile apps. This could be resolved in a new version of the application that stores data locally, with the potential for a data synchronization system to maintain availability of usage data to researchers. We acknowledge the need to adapt the diary for other mobile phone platforms or Web-based application. Finally, taking into account that the MIDAS scores seem inappropriate for day-to-day reporting, it would be desirable to replace this measure with a new one that could examine daily changes. However, more psychometric work is required to develop a new measure.

### Limitations of the Study

This study has some limitations. First, participants across a wide range of ages were included. We encountered recruitment difficulties for participants between 14 and 17 years old, which limits generalizability of our psychometric findings to this age group. Second, introducing a routine such as using this diary every day is neither easy nor fast. When testing usability and feasibility of the diary, we offered participants a prototype version of the diary to use for 14 days. With this time period, which is shorter than the 28-day period that is often recommended for assessment in headache studies [28,29,46], the use of the diary was not effortlessly adapted as daily routine. This could have negatively affected use of the diary, quality of the suggestions, and even bias modifications taken as result of the collected data. A longer trial duration could have minimized these problems and provide a more accurate picture of feasibility data when this diary is used as consistently recommended in the literature. Third, findings from interviews conducted in each cycle of usability/feasibility were not representative of the participating sample in each cycle as only 15%-25% of participants were involved in the interviews. Despite this inherent characteristic of qualitative research, the data helped to provide richer insight of some participant experiences using the diary. Similarly, the case study is not generalizable; however, it provides an example of how this diary may be helpful. Finally, allowing participants to freely omit some items on the questionnaire may result in greater levels of missing data. In a clinical context, practitioners should work with patients closely to determine which questionnaire items are most important to track on a case-by-case basis.

### Conclusions and Next Steps

This research represents the necessary first steps toward creating a feasible and psychometrically sound electronic diary for young adults with headaches. Users should be continuously involved during the design of applications [47] and we involved users from the onset [48]. The application was evaluated using formal testing cycles with participants once we had a high-fidelity and fully functional prototype of the application to provide an accurate and realistic setting for evaluation, following Prinz’s recommendations [49]. This diary may be useful not only for individuals with headaches but also for medical doctors who want to collect accurate and thorough information. Without a diary, medical doctors may be forced to make care decisions on the basis of limited retrospective information collected during brief and sporadic encounters. They will now have an accurate picture of their patients’ headaches in order to tailor headache treatments to each patient, which ultimately may improve treatment outcomes. The diary records of frequency, severity, duration, location, qualities of headaches, the level of disability, and its associated symptoms can help to consolidate headache diagnosis. The exposure to potential triggers and perceived precipitating factors can help identify headache triggers, and its records of frequency of analgesic use and other coping strategies can help to determine the best methods for managing pain.

Since there is still no evidence indicating the multiple potential benefits of electronic diaries in the field of headache [50] our plan for the future is to evaluate the impact of this diary at multiple levels (eg, does the use of myWHI save clinician time? does it facilitate diagnosis? does it improve patient outcomes?). The authors plan to make this diary publically available soon at the myWHI website.

## Abbreviations

DHD: daily headache diary
HA: Headache
MIDAS: Migraine Disability Assessment Scale
Mdn: Median
myWHI diary: myWireless Headache Intervention diary
PROMIS: Patient Reported Outcomes Measurement Information System

## References

[1] Vos Theo, Flaxman Abraham D, Naghavi Mohsen, Lozano Rafael, Michaud Catherine, Ezzati Majid, Shibuya Kenji, Salomon Joshua A, Abdalla Safa, Aboyans Victor, Abraham Jerry, Ackerman Ilana, Aggarwal Rakesh, Ahn Stephanie Y, Ali Mohammed K, Alvarado Miriam, Anderson H Ross, Anderson Laurie M, Andrews Kathryn G, Atkinson Charles, Baddour Larry M, Bahalim Adil N, Barker-Collo Suzanne, Barrero Lope H, Bartels David H, Basáñez Maria-Gloria, Baxter Amanda, Bell Michelle L, Benjamin Emelia J, Bennett Derrick, Bernabé Eduardo, Bhalla Kavi, Bhandari Bishal, Bikbov Boris, Bin Abdulhak Aref, Birbeck Gretchen, Black James A, Blencowe Hannah, Blore Jed D, Blyth Fiona, Bolliger Ian, Bonaventure Audrey, Boufous Soufiane, Bourne Rupert, Boussinesq Michel, Braithwaite Tasanee, Brayne Carol, Bridgett Lisa, Brooker Simon, Brooks Peter, Brugha Traolach S, Bryan-Hancock Claire, Bucello Chiara, Buchbinder Rachelle, Buckle Geoffrey, Budke Christine M, Burch Michael, Burney Peter, Burstein Roy, Calabria Bianca, Campbell Benjamin, Canter Charles E, Carabin Hélène, Carapetis Jonathan, Carmona Loreto, Cella Claudia, Charlson Fiona, Chen Honglei, Cheng Andrew Tai-Ann, Chou David, Chugh Sumeet S, Coffeng Luc E, Colan Steven D, Colquhoun Samantha, Colson K Ellicott, Condon John, Connor Myles D, Cooper Leslie T, Corriere Matthew, Cortinovis Monica, Couser William, Cowie Benjamin C, Criqui Michael H, Cross Marita, Dabhadkar Kaustubh C, Dahiya Manu, Dahodwala Nabila, Damsere-Derry James, Danaei Goodarz, Davis Adrian, De Leo Diego, Degenhardt Louisa, Dellavalle Robert, Delossantos Allyne, Denenberg Julie, Derrett Sarah, Dharmaratne Samath D, Dherani Mukesh, Diaz-Torne Cesar, Dolk Helen, Dorsey E Ray, Driscoll Tim, Duber Herbert, Ebel Beth, Edmond Karen, Elbaz Alexis, Ali Suad Eltahir, Erskine Holly, Erwin Patricia J, Espindola Patricia, Ewoigbokhan Stalin E, Farzadfar Farshad, Feigin Valery, Felson David T, Ferrari Alize, Ferri Cleusa P, Fèvre Eric M, Finucane Mariel M, Flaxman Seth, Flood Louise, Foreman Kyle, Forouzanfar Mohammad H, Franklin Richard, Fransen Marlene, Freeman Michael K, Gabbe Belinda J, Gabriel Sherine E, Gakidou Emmanuela, Ganatra Hammad A, Garcia Bianca, Gaspari Flavio, Gillum Richard F, Gmel Gerhard, Gosselin Richard, Grainger Rebecca, Groeger Justina, Guillemin Francis, Gunnell David, Gupta Ramyani, Haagsma Juanita, Hagan Holly, Halasa Yara A, Hall Wayne, Haring Diana, Haro Josep Maria, Harrison James E, Havmoeller Rasmus, Hay Roderick J, Higashi Hideki, Hill Catherine, Hoen Bruno, Hoffman Howard, Hotez Peter J, Hoy Damian, Huang John J, Ibeanusi Sydney E, Jacobsen Kathryn H, James Spencer L, Jarvis Deborah, Jasrasaria Rashmi, Jayaraman Sudha, Johns Nicole, Jonas Jost B, Karthikeyan Ganesan, Kassebaum Nicholas, Kawakami Norito, Keren Andre, Khoo Jon-Paul, King Charles H, Knowlton Lisa Marie, Kobusingye Olive, Koranteng Adofo, Krishnamurthi Rita, Lalloo Ratilal, Laslett Laura L, Lathlean Tim, Leasher Janet L, Lee Yong Yi, Leigh James, Lim Stephen S, Limb Elizabeth, Lin John Kent, Lipnick Michael, Lipshultz Steven E, Liu Wei, Loane Maria, Ohno Summer Lockett, Lyons Ronan, Ma Jixiang, Mabweijano Jacqueline, MacIntyre Michael F, Malekzadeh Reza, Mallinger Leslie, Manivannan Sivabalan, Marcenes Wagner, March Lyn, Margolis David J, Marks Guy B, Marks Robin, Matsumori Akira, Matzopoulos Richard, Mayosi Bongani M, McAnulty John H, McDermott Mary M, McGill Neil, McGrath John, Medina-Mora Maria Elena, Meltzer Michele, Mensah George A, Merriman Tony R, Meyer Ana-Claire, Miglioli Valeria, Miller Matthew, Miller Ted R, Mitchell Philip B, Mocumbi Ana Olga, Moffitt Terrie E, Mokdad Ali A, Monasta Lorenzo, Montico Marcella, Moradi-Lakeh Maziar, Moran Andrew, Morawska Lidia, Mori Rintaro, Murdoch Michele E, Mwaniki Michael K, Naidoo Kovin, Nair M Nathan, Naldi Luigi, Nelson Paul K, Nelson Robert G, Nevitt Michael C, Newton Charles R, Nolte Sandra, Norman Paul, Norman Rosana, O'Donnell Martin, O'Hanlon Simon, Olives Casey, Omer Saad B, Ortblad Katrina, Osborne Richard, Ozgediz Doruk, Page Andrew, Pahari Bishnu, Pandian Jeyaraj Durai, Rivero Andrea Panozo, Patten Scott B, Pearce Neil, Padilla Rogelio Perez, Perez-Ruiz Fernando, Perico Norberto, Pesudovs Konrad, Phillips David, Phillips Michael R, Pierce Kelsey, Pion Sébastien, Polanczyk Guilherme V, Polinder Suzanne, Pope C Arden, Popova Svetlana, Porrini Esteban, Pourmalek Farshad, Prince Martin, Pullan Rachel L, Ramaiah Kapa D, Ranganathan Dharani, Razavi Homie, Regan Mathilda, Rehm Jürgen T, Rein David B, Remuzzi Guiseppe, Richardson Kathryn, Rivara Frederick P, Roberts Thomas, Robinson Carolyn, Ronfani Luca, Room Robin, Rosenfeld Lisa C, Rushton Lesley, Sacco Ralph L, Saha Sukanta, Sampson Uchechukwu, Sanchez-Riera Lidia, Sanman Ella, Schwebel David C, Scott James Graham, Segui-Gomez Maria, Shahraz Saeid, Shepard Donald S, Shin Hwashin, Shivakoti Rupak, Singh David, Singh Gitanjali M, Singh Jasvinder A, Singleton Jessica, Sleet David A, Sliwa Karen, Smith Emma, Smith Jennifer L, Steer Andrew, Steiner Timothy, Stolk Wilma A, Stovner Lars Jacob, Sudfeld Christopher, Syed Sana, Tamburlini Giorgio, Tavakkoli Mohammad, Taylor Hugh R, Taylor Jennifer A, Taylor William J, Thomas Bernadette, Thomson W Murray, Thurston George D, Tleyjeh Imad M, Tonelli Marcello, Towbin Jeffrey A, Truelsen Thomas, Tsilimbaris Miltiadis K, Ubeda Clotilde, Undurraga Eduardo A, van Os Jim, Vavilala Monica S, Venketasubramanian N, Wang Mengru, Wang Wenzhi, Watt Kerrianne, Weatherall David J, Weinstock Martin A, Weintraub Robert, Weisskopf Marc G, Weissman Myrna M, White Richard A, Whiteford Harvey, Wiersma Steven T, Wilkinson James D, Williams Hywel C, Witt Emma, Wolfe Frederick, Woolf Anthony D, Wulf Sarah, Yeh Pon-Hsiu, Zheng Zhi-Jie, Zonies David, Lopez Alan D, AlMazroa Mohammad A, Memish Ziad A, de Vaccaro Karen Courville, Des Jarlais Don C, Fowkes Francis Gerry R, Narayan K M Venkat, De Leòn Felipe Rodriguez, Stapelberg Nicolas J C, van der Werf Marieke J, Williams Sean R M, Zaidi Anita K M, Murray Christopher J L (2012). Years lived with disability (YLDs) for 1160 sequelae of 289 diseases and injuries 1990-2010: a systematic analysis for the Global Burden of Disease Study 2010. Lancet.

[2] Headache Classification Committee of the International Headache Society (IHS) (2013). The International Classification of Headache Disorders, 3rd edition (beta version). Cephalalgia.

[3] Dowson A J, Sender J, Lipscombe S, Cady R K, Tepper S J, Smith R, Smith T R, Taylor F R, Boudreau G P, Poole A C, Baos V, Wöber C, van Duijn N P (2003). Establishing principles for migraine management in primary care. Int J Clin Pract.

[4] Peng Kuan-Po, Wang Shuu-Jiun (2012). Migraine diagnosis: screening items, instruments, and scales. Acta Anaesthesiol Taiwan.

[5] Winkler R, Underwood P, Fatovich B, James R, Gray D (1989). A clinical trial of a self-care approach to the management of chronic headache in general practice. Soc Sci Med.

[6] Baldacci Filippo, Vedovello Marcella, Ulivi Martina, Vergallo Andrea, Poletti Michele, Borelli Paolo, Nuti Angelo, Bonuccelli Ubaldo (2013). How aware are migraineurs of their triggers?. Headache.

[7] Moloney Margaret F, Aycock Dawn M, Cotsonis George A, Myerburg Stuart, Farino Christopher, Lentz Martha (2009). An Internet-based migraine headache diary: issues in Internet-based research. Headache.

[8] Nicholson Robert A, Buse Dawn C, Andrasik Frank, Lipton Richard B (2011). Nonpharmacologic treatments for migraine and tension-type headache: how to choose and when to use. Curr Treat Options Neurol.

[9] WHO (2006). Neurological Disorders: Public Health Challenges.

[10] Martin Paul R (2010). Behavioral management of migraine headache triggers: learning to cope with triggers. Curr Pain Headache Rep.

[11] Huguet Anna, McGrath Patrick J, Stinson Jennifer, Tougas Michelle E, Doucette Steve (2014). Efficacy of psychological treatment for headaches: an overview of systematic reviews and analysis of potential modifiers of treatment efficacy. Clin J Pain.

[12] Connelly Mark, Bickel Jennifer (2011). An electronic daily diary process study of stress and health behavior triggers of primary headaches in children. J Pediatr Psychol.

[13] Bandell-Hoekstra E N, Abu-Saad H H, van den Brink M (2001). The occurrence of recall bias in pediatric headache: a comparison of questionnaire and diary data. Headache.

[14] Sorbi Marjolijn J, Mak Sander B, Houtveen Jan H, Kleiboer Annet M, van Doornen Lorenz J P (2007). Mobile Web-based monitoring and coaching: feasibility in chronic migraine. J Med Internet Res.

[15] Stinson Jennifer N, Jibb Lindsay A, Nguyen Cynthia, Nathan Paul C, Maloney Anne Marie, Dupuis L Lee, Gerstle J Ted, Alman Benjamin, Hopyan Sevan, Strahlendorf Caron, Portwine Carol, Johnston Donna L, Orr Mike (2013). Development and testing of a multidimensional iPhone pain assessment application for adolescents with cancer. J Med Internet Res.

[16] Palermo Tonya M, Valenzuela Duaré, Stork Paul P (2004). A randomized trial of electronic versus paper pain diaries in children: impact on compliance, accuracy, and acceptability. Pain.

[17] Stone Arthur A, Shiffman Saul (2002). Capturing momentary, self-report data: a proposal for reporting guidelines. Ann Behav Med.

[18] Stone Arthur A, Shiffman Saul, Schwartz Joseph E, Broderick Joan E, Hufford Michael R (2003). Patient compliance with paper and electronic diaries. Control Clin Trials.

[19] Affleck G, Zautra A, Tennen H, Armeli S (1999). Multilevel daily process designs for consulting and clinical psychology: a preface for the perplexed. J Consult Clin Psychol.

[20] Burton Christopher, Weller David, Sharpe Michael (2007). Are electronic diaries useful for symptoms research? A systematic review. J Psychosom Res.

[21] Lewandowski Amy S, Palermo Tonya M, Kirchner H Lester, Drotar Dennis (2009). Comparing diary and retrospective reports of pain and activity restriction in children and adolescents with chronic pain conditions. Clin J Pain.

[22] Stinson Jennifer N, Huguet Anna, McGrath Patrick, Rosenbloom Brittany, Soobiah Charlene, White Meghan, Coburn Geraldine (2013). A qualitative review of the psychometric properties and feasibility of electronic headache diaries for children and adults: where we are and where we need to go. Pain Res Manag.

[23] Hundert Amos S, Huguet Anna, McGrath Patrick J, Stinson Jennifer N, Wheaton Mike (2014). Commercially available mobile phone headache diary apps: a systematic review. JMIR Mhealth Uhealth.

[24] Skeels Meredith M, Pratt Wanda (2008). Participatory design with health consumers. AMIA Annu Symp Proc.

[25] Dworkin Robert H, Turk Dennis C, Farrar John T, Haythornthwaite Jennifer A, Jensen Mark P, Katz Nathaniel P, Kerns Robert D, Stucki Gerold, Allen Robert R, Bellamy Nicholas, Carr Daniel B, Chandler Julie, Cowan Penney, Dionne Raymond, Galer Bradley S, Hertz Sharon, Jadad Alejandro R, Kramer Lynn D, Manning Donald C, Martin Susan, McCormick Cynthia G, McDermott Michael P, McGrath Patrick, Quessy Steve, Rappaport Bob A, Robbins Wendye, Robinson James P, Rothman Margaret, Royal Mike A, Simon Lee, Stauffer Joseph W, Stein Wendy, Tollett Jane, Wernicke Joachim, Witter James, IMMPACT (2005). Core outcome measures for chronic pain clinical trials: IMMPACT recommendations. Pain.

[26] McGrath Patrick J, Walco Gary A, Turk Dennis C, Dworkin Robert H, Brown Mark T, Davidson Karina, Eccleston Christopher, Finley G Allen, Goldschneider Kenneth, Haverkos Lynne, Hertz Sharon H, Ljungman Gustaf, Palermo Tonya, Rappaport Bob A, Rhodes Thomas, Schechter Neil, Scott Jane, Sethna Navil, Svensson Ola K, Stinson Jennifer, Walker Lynn, Weisman Steven, White Richard E, Zajicek Anne, Zeltzer Lonnie, von Baeyer Carl L, PedIMMPACT (2008). Core outcome domains and measures for pediatric acute and chronic/recurrent pain clinical trials: PedIMMPACT recommendations. J Pain.

[27] Turk Dennis C, Dworkin Robert H, Allen Robert R, Bellamy Nicholas, Brandenburg Nancy, Carr Daniel B, Cleeland Charles, Dionne Raymond, Farrar John T, Galer Bradley S, Hewitt David J, Jadad Alejandro R, Katz Nathaniel P, Kramer Lynn D, Manning Donald C, McCormick Cynthia G, McDermott Michael P, McGrath Patrick, Quessy Steve, Rappaport Bob A, Robinson James P, Royal Mike A, Simon Lee, Stauffer Joseph W, Stein Wendy, Tollett Jane, Witter James (2003). Core outcome domains for chronic pain clinical trials: IMMPACT recommendations. Pain.

[28] Bendtsen L, Bigal M E, Cerbo R, Diener H C, Holroyd K, Lampl C, Mitsikostas D D, Steiner T J, Tfelt-Hansen P, International Headache Society Clinical Trials Subcommittee (2010). Guidelines for controlled trials of drugs in tension-type headache: second edition. Cephalalgia.

[29] Penzien Donald B, Andrasik Frank, Freidenberg Brian M, Houle Timothy T, Lake Alvin E, Lipchik Gay L, Holroyd Kenneth A, Lipton Richard B, McCrory Douglas C, Nash Justin M, Nicholson Robert A, Powers Scott W, Rains Jeanetta C, Wittrock David A, American Headache Society Behavioral Clinical Trials Workgroup (2005). Guidelines for trials of behavioral treatments for recurrent headache, first edition: American Headache Society Behavioral Clinical Trials Workgroup. Headache.

[30] Tfelt-Hansen P, Block G, Dahlöf C, Diener H C, Ferrari M D, Goadsby P J, Guidetti V, Jones B, Lipton R B, Massiou H, Meinert C, Sandrini G, Steiner T, Winter P B, International Headache Society Clinical Trials Subcommittee (2000). Guidelines for controlled trials of drugs in migraine: second edition. Cephalalgia.

[31] Spagrud Lara J, McCormick Julia C, Choo Eugene, Neville Kathleen, Connelly Mark A, von Baeyer Carl L (2009). Three new datasets supporting use of the Numerical Rating Scale (NRS-11) for children's self-reports of pain intensity. Pain.

[32] Jensen M P, Turner J A, Romano J M, Fisher L D (1999). Comparative reliability and validity of chronic pain intensity measures. Pain.

[33] Jensen M P, Karoly P, Braver S (1986). The measurement of clinical pain intensity: a comparison of six methods. Pain.

[34] Berwick D M, Murphy J M, Goldman P A, Ware J E, Barsky A J, Weinstein M C (1991). Performance of a five-item mental health screening test. Med Care.

[35] Rumpf H J, Meyer C, Hapke U, John U (2001). Screening for mental health: validity of the MHI-5 using DSM-IV Axis I psychiatric disorders as gold standard. Psychiatry Res.

[36] Strand Bjørn Heine, Dalgard Odd Steffen, Tambs Kristian, Rognerud Marit (2003). Measuring the mental health status of the Norwegian population: a comparison of the instruments SCL-25, SCL-10, SCL-5 and MHI-5 (SF-36). Nord J Psychiatry.

[37] Buysse Daniel J, Yu Lan, Moul Douglas E, Germain Anne, Stover Angela, Dodds Nathan E, Johnston Kelly L, Shablesky-Cade Melissa A, Pilkonis Paul A (2010). Development and validation of patient-reported outcome measures for sleep disturbance and sleep-related impairments. Sleep.

[38] Amtmann Dagmar, Cook Karon F, Jensen Mark P, Chen Wen-Hung, Choi Seung, Revicki Dennis, Cella David, Rothrock Nan, Keefe Francis, Callahan Leigh, Lai Jin-Shei (2010). Development of a PROMIS item bank to measure pain interference. Pain.

[39] Wolfson A R, Carskadon M A (1998). Sleep schedules and daytime functioning in adolescents. Child Dev.

[40] Headache Classification Subcommittee of the International Headache Society (2004). The International Classification of Headache Disorders: 2nd edition. Cephalalgia.

[41] Varni James W, Stucky Brian D, Thissen David, Dewitt Esi Morgan, Irwin Debra E, Lai Jin-Shei, Yeatts Karin, Dewalt Darren A (2010). PROMIS Pediatric Pain Interference Scale: an item response theory analysis of the pediatric pain item bank. J Pain.

[42] Braun V, Clarke V (2006). Using thematic analysis in psychology. Qualitative Research in Psychology.

[43] Cranford James A, Shrout Patrick E, Iida Masumi, Rafaeli Eshkol, Yip Tiffany, Bolger Niall (2006). A procedure for evaluating sensitivity to within-person change: can mood measures in diary studies detect change reliably?. Pers Soc Psychol Bull.

[44] Cohen J (1988). Statistical power analysis for the behavioral sciences.

[45] Allena Marta, Cuzzoni Maria Giovanna, Tassorelli Cristina, Nappi Giuseppe, Antonaci Fabio (2012). An electronic diary on a palm device for headache monitoring: a preliminary experience. J Headache Pain.

[46] Silberstein S, Tfelt-Hansen P, Dodick D W, Limmroth V, Lipton R B, Pascual J, Wang S J, Task Force of the International Headache Society Clinical Trials Subcommittee (2008). Guidelines for controlled trials of prophylactic treatment of chronic migraine in adults. Cephalalgia.

[47] Lynch T, Gregor S (2004). User participation in decision support systems development: Influencing system outcomes. Eur J Inf Syst.

[48] Huguet Anna, Stinson Jennifer, Mackay Bonnie, Watters Carolyn, Tougas Michelle, White Meghan, McGrath Patrick J (2014). Bringing psychosocial support to headache sufferers using information and communication technology: lessons learned from asking potential users what they want. Pain Res Manag.

[49] Prinz W, Mark G, Pankoke-Babatz U (1998). Designing groupware for congruency in use. CSCW '98: computer supported cooperative work: ACM 1998 Conference on Computer Supported Cooperative Work: proceedings, Seattle, Washington, November 14-18, 1998.

[50] Marceau Lisa D, Link Carol, Jamison Robert N, Carolan Sarah (2007). Electronic diaries as a tool to improve pain management: is there any evidence?. Pain Med.

